# Pollination triggers female gametophyte development in immature *Nicotiana tabacum* flowers

**DOI:** 10.3389/fpls.2015.00561

**Published:** 2015-07-22

**Authors:** Michael S. Brito, Lígia T. Bertolino, Viviane Cossalter, Andréa C. Quiapim, Henrique C. DePaoli, Gustavo H. Goldman, Simone P. Teixeira, Maria H. S. Goldman

**Affiliations:** ^1^Departamento de Biologia, Faculdade de Filosofia, Ciências e Letras de Ribeirão Preto, Universidade de São PauloRibeirão Preto, Brazil; ^2^Programa de Pós-Graduação em Genética, Faculdade de Medicina de Ribeirão Preto, Universidade de São PauloRibeirão Preto, Brazil; ^3^Programa de Pós-Graduação em Genética e Melhoramento de Plantas, Faculdade de Ciências Agrárias e Veterinárias, Universidade Estadual Paulista “Júlio de Mesquita Filho,”Jaboticabal, Brazil; ^4^Departamento de Ciências Farmacêuticas, Faculdade de Ciências Farmacêuticas de Ribeirão Preto, Universidade de São PauloRibeirão Preto, Brazil

**Keywords:** stigma receptivity, pollen tube growth, pollination signal, female gametophyte development, fruit weight, seed germination capacity

## Abstract

In *Nicotiana tabacum*, female gametophytes are not fully developed at anthesis, but flower buds pollinated 12 h before anthesis produce mature embryo sacs. We investigated several pollination-associated parameters in *N. tabacum* flower buds to determine the developmental timing of important events in preparation for successful fertilization. First, we performed hand pollinations in flowers from stages 4 to 11 to study at which developmental stage pollination would produce fruits. A Peroxtesmo test was performed to correlate peroxidase activity on the stigma surface, indicative of stigma receptivity, with fruit set. Pollen tube growth and female gametophyte development were microscopically analyzed in pistils of different developmental stages. Fruits were obtained only after pollinations of flower buds at late stage 7 and older; fruit weight and seed germination capacity increased as the developmental stage of the pollinated flower approached anthesis. Despite positive peroxidase activity and pollen tube growth, pistils at stages 5 and 6 were unable to produce fruits. At late stage 7, female gametophytes were undergoing first mitotic division. After 24 h, female gametophytes of unpollinated pistils were still in the end of the first division, whereas those of pollinated pistils showed egg cells. RT-qPCR assay showed that the expression of the *NtEC1* gene, a marker of egg cell development, is considerably higher in pollinated late stage 7 ovaries compared with unpollinated ovaries. To test whether ethylene is the signal eliciting female gametophyte maturation, the expression of ACC synthase was examined in unpollinated and pollinated stage 6 and late stage 7 stigmas/styles. Pollination induced *NtACS* expression in stage 6 pistils, which are unable to produce fruits. Our results show that pollination is a stimulus capable of triggering female gametophyte development in immature tobacco flowers and suggests the existence of a yet undefined signal sensed by the pistil.

## Introduction

Angiosperms correspond to a group of plants with distinct characteristics, including the presence of ovules enclosed in a maternal organ, know as the pistil. The pistil is generally composed of a stigma, a style, and an ovary that develops into a fruit after fertilization. The fertilization process begins with the deposition of pollen grains onto a receptive stigma surface. When pollen grain recognition and acceptance occurs, it will hydrate and germinate, producing a pollen tube that grows through the style in the direction of the ovary until reaching an embryo sac, known as the angiosperm female gametophyte. Next, the pollen tube bursts, releasing two sperm cells in the interior of the embryo sac. One of the sperm cells fuses to the egg cell to produce a diploid embryo, whereas the second sperm cell fuses to the central cell, generating a triploid endosperm (Lersten, 2004).

Successful reproduction in angiosperms depends on a series of cell–cell interactions between male gametophytes and the specialized tissues of the pistil and female gametophytes (Beale and Johnson, 2013). Over the last few years, new information and discoveries have increased our knowledge about the signals produced by the female gametophytes to attract and direct the pollen tubes (Beale and Johnson, 2013). However, less is known about signals produced by the pollen tube or pistil in response to pollen tube growth, establishing communication with female gametophytes. Some evidence of this male–female directional signaling comes from studies of orchid species, in which ovule differentiation and development are pollination-dependent (Zhang and O’Neill, 1993; O’Neill, 1997). Once inside the ovule, the orchid pollen tube waits for the female gametophyte to complete development before releasing sperm cells to promote fertilization (O’Neill et al., 1993). In other plants, such as almond, the ovule is partially developed at anthesis and reaches full maturation only after pollination (Pimienta and Polito, 1983). This pattern of female gametophyte development triggered by pollination stimulus has also been observed for sweet pepper (Ofosu-Anim et al., 2006) and maize (Mól et al., 2000).

In breeding programs, researchers may perform bud pollination to overcome incongruity in interspecific crosses or self-incompatibility. Hand pollination using mature pollen can be performed on immature flowers (Haring et al., 1990) in an attempt to bypass the effects of self-incompatibility on species of Brassicaceae (Hiscock and Dickinson, 1993), as well as Solanaceae species (Chalivendra et al., 2013). In *Nicotiana tabacum*, a species with a Polygonum-type embryo sac (Huang and Russell, 1992), the ovule is not fully developed at anthesis, and the egg cell is not usually observed at the embryo sacs (Tian and Russell, 1997; Lobanova and Enaleeva, 1998; De Martinis and Mariani, 1999; Chen et al., 2012). In this species, the effects of pollination on ovule development have mainly been examined at stages close to anthesis. Hand-pollinated tobacco flower buds 12 h before anthesis reach the mature embryo sac stage earlier than flower buds emasculated and not pollinated (Tian and Russell, 1997), suggesting the existence of a male–female directional signaling. De Martinis and Mariani (1999) noted that pollinations in young flower buds (stage 6) do not induce embryo sac formation and seed production, but they did not investigate this aspect in detail. Thus, little is known regarding the developmental timing of important events in preparation for successful fertilization in *N. tabacum* flowers.

Our hypothesis is that pollinations performed on young flower buds will be effective and produce fruits, despite the fact that *N. tabacum* female gametophytes are not fully developed at anthesis. At which flower developmental stage pollination will be productive? We have examined several parameters related to reproductive success (Calixto et al., 2009), such as fruit formation, seed production and germination capacity, and correlated them with stigma receptivity based on peroxidase activity, microscopy analysis of pollen tube growth, embryo sac development, and *NtEC1* (*Egg Cell 1*) gene expression. Our results represent a detailed analysis of the effects of pollination on *N. tabacum* flower buds at stages prior to anthesis and shows that preparation for successful fertilization is a gradual process in which the necessary requirements are achieved in phases. This work provides evidence for the existence of male–female signaling produced by pollination and considers whether ethylene could be this signal through an investigation of ACC synthase expression. We have shown that a yet undefined pollination signal is sensed by the *N. tabacum* pistil throughout half of its development and is sufficient to trigger cellular and molecular female gametophyte maturation.

## Materials and Methods

### Plant Material

Seeds from *N. tabacum* cv. Petit Havana SR1 were sown in expanded polystyrene trays containing PlantMax commercial substrate (Eucatex, Brazil). After germination and growth to a height of approximately 3 cm, plantlets were transferred to plastic bags and later to 20 L vases. During germination and growth, plants were cultivated in standard greenhouse conditions and irrigated by aspersion. The stages of tobacco flower development were determined using parameters previously described by Koltunow et al. (1990).

### Controlled Pollinations and Fruit Analyses

Tobacco pistils from stages 4 to 11 of flower development were emasculated and hand pollinated with mature pollen grains from flowers at anthesis (stage 12). Stage 12 flowers were not included in this work because they are naturally pollinated at this stage. For each analyzed stage, a minimum of eight pistils (from at least six independent plants) were hand pollinated and labeled with sewing threads of different colors. Approximately 20 days after pollination, the pollinated pistils were analyzed for the presence or absence of fruits. The obtained fruits were collected individually and dried at room temperature for approximately 2 days. On the third day, fruits were separately weighed on a precision balance (Acculab – L series). The data obtained were analyzed statistically using an analysis of variance (ANOVA) of PROC GLM (software SAS version 9). When variation between two stages was detected, differences with *p* < 0.05 were considered significant.

### Analysis of Seed Germination

To establish the germination capacity of the seeds produced, 300 seeds (from fruits obtained at each developmental stage) were placed in sterile wet filter paper (100 seeds per plate). Two weeks later, the number of germinated seeds was counted, and the results were analyzed using Student’s *t*-test (*p* ≤ 0.05).

### Determination of Stigma Receptivity

To study the stigma receptivity, we used special peroxidase test papers (Peroxtesmo KO, Macherey-Nagel – Düren, Germany) as proposed by Dafni and Maués (1998). For this purpose, four stigmas from tobacco flowers at stages 4 to 11 were pressed against peroxidase test-paper and were regarded as positive when blue coloration developed.

### Analyses of Pollen Tube Growth

Controlled hand pollinations were performed with stages 4–11 tobacco pistils, as described above. According to De Graaf et al. (2003), pollen tubes reach the tobacco ovary 24 h after pollination. Thus, pollinated pistils were excised 24 h after hand pollination. Stigmas/styles and ovaries were separated and immediately fixed in FPA 50 [2.5 mL of 37% formaldehyde (Sigma), 2.5 mL of propionic acid (Vetec – Brazil), and 45 mL of 50% ethanol (Merck)]. The samples were subjected to 15 mmHg vacuum for 15 min in the presence of the fixative. This procedure was repeated four times, and the material was left on the fixative overnight. The fixative was substituted by 50% ethanol, and the material was incubated at 8°C overnight. The next day, 50% ethanol was substituted by 70% ethanol. Longitudinally hand-opened stigmas/styles and ovaries were placed on a glass slide and stained in a 0.1% solution of aniline blue in 0.1 N K_3_PO_4_ (Kho and Bera, 1968). The samples were carefully squashed between a glass slide and coverslip in the aniline blue solution, revealing the pollen tube callose plugs. Visualization and documentation were performed with a Zeiss Axiolab epifluorescence microscope (HBO 103W/2 lamp) using an excitation wavelength of 450/90 nm and an emission wavelength of 520 nm. Images were taken using a Zeiss AxioCam Color 412-312 and AxioVision LE4.8 software.

### Microscopic Analyses of Pollinated and Unpollinated Ovaries

Late stage 7 flowers (35 mm) were emasculated and kept unpollinated or were pollinated with pollen grains from stage 12 flowers (anthesis). Ovaries from both pollinated and unpollinated flowers were harvested after 24 h and fixed in FAA 50 [5 mL of glacial acetic acid (Merck), 5 mL of 37% formaldehyde (Sigma), and 90 mL of 50% ethanol (Merck)] for 24 h (Johansen, 1940). Then, the samples were transferred to 50% ethanol and subsequently to 70% ethanol, in which they were stored. The ovaries were dehydrated in a graded ethanol/xylol series and embedded in paraffin. The embedded material was sliced into 6-μm sections, mounted on microscope slides and stained with 0.05% of toluidine blue pH 6.8. The pictures were taken using a Leica DM50 microscope equipped with a Leica DFC 320 digital camera.

### RNA Extraction, cDNA Synthesis, and RT-qPCR Analysis

As described above, stage 6 and late stage 7 flowers were emasculated and kept unpollinated or were pollinated with mature pollen. After 24 h, stigmas/styles and ovaries were collected in liquid nitrogen and stored at -80°C (three biological replicates for each condition, each replicate containing three pistil samples). The RNA of each sample was extracted using Trizol (Invitrogen^®^) according to the manufacturer’s protocol. RNA integrity was checked by electrophoresis in 1.2% agarose and 20 mM guanidine isothiocyanate gel. RNA samples were treated with RNase-free DNase (Promega^®^) following the manufacturer’s instructions and an aliquot was used to check for genomic DNA contamination in a standard qPCR with GAPDH (glyceraldehyde 3-phosphate dehydrogenase) primers (see below). DNA-free RNA was cleaned using *Clean up – Rneasy Mini Kit* (Qiagen) and quantified in a NanoDrop 2000 (Thermo Scientific). SuperScript III reverse transcriptase (Invitrogen^®^) was used to generate cDNA from 1 μg of the RNA samples. qPCR experiments were carried out in three technical replicates on an Applied Biosystems 7500 Fast Real-Time PCR System. Each reaction was composed of 5 μL of GoTaq qPCR Master Mix (Promega), 1 μL of sterile Milli-Q purified water, 1 μL (2.5 μM) of each adequate primer (GAPDH forward GCATCTTTGATGCCAAGGCTGGAA and GAPDH reverse TCGAGTGCTGTAGCCCATTTCGTT; RPL2 forward CGGGTGTGTCACTTTCCGTTACCCG and reverse ATACCCTCAGCAGCCACGAAC; NtEC1 forward CTGTTGGCCTTCTATGCTTACT and reverse GGTTGAGGTGATGGAGTTC; and NtACS forward TTCAGAGCCTGGTTGGTTTAG and reverse GACTCCTCCTTCAATCCCTTTAC), and 2 μL of cDNA. The cycling conditions consisted of a initial step of 50°C for 2 min and 95°C for 10 min, followed by 40 cycles of 95°C for 15 s and 60°C for 1 min. RPL2 (Ribosomal Protein L2) and GAPDH were previously validated by our group as the best reference genes for different experimental conditions and pistil samples (unpublished results). The efficiency of primer pairs was determined from the slope of the standard curve using the formula Efficiency (*E*) = 10 (-1/slope) and then converted to percentage efficiency, where % of efficiency = (*E-*1) × 100%. Confirmation of amplicon specificity was based on the dissociation curve at the end of each run (ramp time 55–95°C). qPCR reactions in the absence of template were also performed as negative controls for each pair of primers. The expression levels of *NtEC1* and *NtACS* were determined using the formula: 2-ΔCt, where ΔCt = (Cttag – Ctref), Ct = threshold cycle, tag = tag gene, and ref = reference gene, derived from the 2-ΔΔCt method originally published by Livak and Schmittgen (2001). Relative expression was determined by comparing the *NtEC1* transcript expression level between unpollinated, considered as 1, and pollinated ovaries. For *NtACS*, the expression ratios were determined comparing pollinated with unpollinated pistils for each stage (6 or late 7). Statistical analysis using expression data were performed using the REST tool (Pfaﬄ et al., 2002) and data are available in Supplementary Table S1. The accession numbers are: GAPDH – KR007670; RPL2 – X62500; NtEC1 – KP987452; NtACS – X98492.

## Results

### *Nicotiana tabacum* Fruit Weight and Size are Dependent on the Flower Developmental Stage in Which Pollination Occurs

**FIGURE 1:**
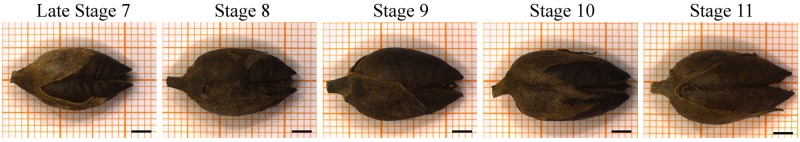
**Representative fruits produced as a result of hand pollinations performed at different stages of *Nicotiana tabacum* flower development.** There is a gradual increase in fruit size from late stage 7 to stage 11.

Controlled pollinations with mature pollen (see Materials and Methods) were performed on stigmas at stages 4–11 of flower development. Pollinations performed on stigmas at stages 4–6 did not produce fruits (**Table 1**). Fruit formation was only observed as a result of pollinations performed at later stages (7–12). However, although fruits were produced in 100% of pollinations performed at stages 8–12, at stage 7, fruit formation was dependent on the specific size of the flower bud (**Table 1**). Fruits were produced only when the pollinated flower buds were 34 mm or longer. Therefore, we divided the stage 7 initially described by Koltunow et al. (1990) into early stage 7, with flower buds with sizes between 28 and 33 mm, and late stage 7, with flower buds with sizes between 34 and 38 mm. Fruits obtained by this analysis were weighted and photographed. The mean fruit weight increased in accordance with the flower developmental stage in which pollination was performed, i.e., the fruit was heavier at later stages (**Table 1**). ANOVA and contrast comparison statistical analyses were performed and demonstrated no significant difference in relation to the mean fruit weight produced among pollinations performed at late stage 7 and stages 8 and 9 (**Table 1**). No statistically significant differences were observed in the mean fruit weight obtained by pollinations at stages 8, 9, 10, and 11 (**Table 1**). However, the mean fruit weight corresponding to late stage 7 was significantly different from the mean fruit weight of pollinations conducted at stages 10 and 11 (**Table 1**). **Figure 1** shows that in addition to fruit weight, fruit size was also influenced by pollinations performed in flower buds at different developmental stages, with size increasing from late stage 7 to stage 11 (**Figure 1**) in parallel with their increasing fruit weight. The most likely explanation for the differences observed in fruit size and weight is the number of seeds successfully produced as a result of pollinations conducted at the different developmental stages.

**Table 1:** Analysis of fruit formation as a result of hand pollinations performed at different stages of *N. tabacum* flower development.

Flower developmental stages	Length of floral bud (mm)^∗^	Number of flower buds pollinated	Presence of fruit	Amount of fruits formed (%)	Average fruit weight (mg)^∗∗^
4	16–19	15	No	0 (0%)	-
5	20–21	15	No	0 (0%)	-
6	22–27	16	No	0 (0%)	-
Early 7	28–33	09	No	0 (0%)	-
Late 7	34–38	11	Yes	11 (100%)	5.88 ± 2.9^a^
8	39–42	17	Yes	17 (100%)	9.20 ± 4.9^ab^
9	43–44	12	Yes	12 (100%)	12.13 ± 4.7^ab^
10	45–46	13	Yes	13 (100%)	14.13 ± 7.6^b^
11	47	12	Yes	12 (100%)	14.75 ± 7.8^b^

*Data originally published by Koltunow et al. (1990) and used in the present work to establish the stages of tobacco flower development.

**Different letters indicate significant differences of fruit weight. Statistical analyses were performed using analysis of variance (ANOVA) and Contrast comparison with *p* ≤ 0.05.

### Pollination at Earlier Flower Developmental Stages Affects Seed Germination Capacity

**FIGURE 2:**
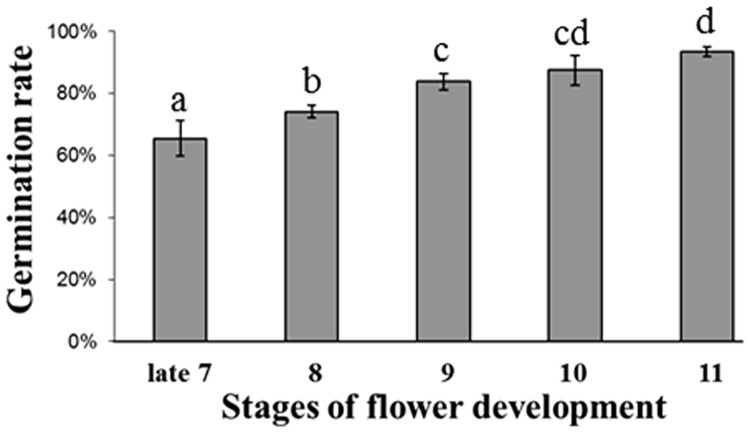
**Tobacco seed germination capacity evaluated as the percentage of germinated seeds.** For each developmental stage, three replicates of 100 seeds each were used. Bars indicate the SD, and different letters represent statistically significant differences by Student’s *t*-test (*p* ≤ 0.05).

To study the germination capacity of seeds produced by pollinations at different developmental stages, we used triplicates of 100 seeds from fruits produced at late stage 7 and onward and placed them in wet filter paper. Two weeks later, the germinated seeds were counted, and the numbers were statistically analyzed (Student’s *t*-test with *p* ≤ 0.05). Seeds from late stage 7 pollinations showed the lowest germination capacity (65% ± 5.7%). As shown in **Figure 2**, the germination capacity increased among seeds produced by pollinations at stage 8 and later toward anthesis. The highest germination capacity (94% ± 1.5%) was verified with seeds of stage 11 pollinations, the latest stage analyzed in this study. Significant differences were observed in the germination capacity of seeds from fruits produced at all developmental stages, except in seeds from stages 9/10, and 10/11 fruits (**Figure 2**). There were seeds capable of germination in all fruits obtained by controlled hand pollinations. However, under our experimental conditions, not all seeds produced were able to germinate, suggesting they were malformed, or physiologically immature.

### Peroxidase Activity Correlates with Stigma Ability to Sustain Pollen Tube Growth

According to Dafni and Maués (1998), the Peroxtesmo KO peroxidase test is the most reliable method for establishing stigma receptivity. Therefore, we have used this test on four stigmas of each flower developmental stage (from 4 to 11) of *N. tabacum*. The peroxidase activity test was negative in all stage 4 stigmas analyzed. In contrast, all stigmas from stages 5 and later showed positive results on the peroxidase activity test, suggesting that stigmas were receptive to pollen grains at developmental stages earlier than anthesis.

**FIGURE 3:**
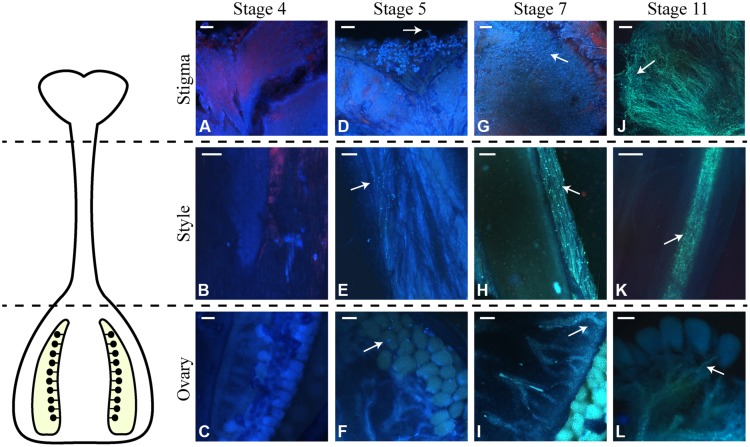
**Germination of pollen grains and pollen tube growth after pollinations performed at different stages of *N. tabacum* flower development.** Pistils of the indicated stages were hand pollinated, and 24 h later, stained with aniline blue and observed by fluorescence microscopy. On the left side of the figure, there is a scheme of the *N. tabacum* pistil, which shows the pistil part observed in each line of the microscopy images (stigma, style, and ovary). **(A)** Pollen grains not germinated on the stigma surface of flowers at stage 4. **(B)** Style of stage 4 flowers in the absence of pollen tubes. **(C)** Ovary of stage 4 flowers where no pollen tube has reached. **(D)** Pollen grains germinated at the stigma surface of stage 5 flowers. **(E)** Pollen tubes growing in the style of stage 5 flowers. **(F)** Pollen tube reaching the ovule (arrow) of stage 5 flowers. **(G)** Pollen grains germinated at the stigma surface of stage 7 flowers. **(H)** Pollen tubes growing in the style of stage 7 flowers. Observe the higher number of pollen tubes compared to the stage 5 style. **(I)** Detail of ovules of stage 7 flowers, where pollen tubes have reached. **(J)** Pollen grains germinated at the stigma surface of stage 11 flowers. **(K)** Growth of pollen tubes in the style of stage 11 flowers. There are more pollen tubes when compared to stages 7 and 5. **(L)** Detail of the ovules present inside the ovary of stage 11 flowers. Arrows indicate pollen tubes. Scale bars represent 60 μm in **(A,B,D,E,G,H,J,K,L)**; 150 μm in **(C,F,I)**.

Effective stigma receptivity was assessed by the ability to sustain pollen germination and pollen tube growth. For this purpose, pistils from different development stages (4 to 11) were hand pollinated with mature pollen grains obtained from open flowers (stage 12). Pistils were collected 24 h after pollination, a period of time sufficient for pollen tubes to reach the ovary (De Graaf et al., 2003). After aniline blue staining, the pollen tubes were observed under fluorescence microscopy. In pollinations performed on stage 4 pistils, the pollen grains tended not to remain on the stigma surface, and no pollen hydration was observed. Consequently, no growing pollen tubes were detected on the stigma or style or at the ovary (**Figures 3A–C**). For pollinations performed on pistils of stage 5 flower buds, the pollen grains on the stigma surface hydrated and emitted pollen tubes (**Figures 3D,E**). It was also possible to visualize pollen tubes growing through the entire style length, reaching the ovary (**Figure 3F**) and ovules (Supplementary Figure S1). Pollinations performed at stage 6 and later resulted in an increasing number of hydrated pollen grains and pollen tubes growing through the style. This is clearly shown for the pollination of stages 7 and 11 pistils, shown in **Figures 3G–L**. Therefore, the inability to produce fruits in pollinations performed in stages 5 and 6 pistils is not due to incompetence of the stigmas/styles to sustain pollen tube growth.

### Pollination Stimulates *N. tabacum* Ovule Maturation Prior to Anthesis

**FIGURE 4:**
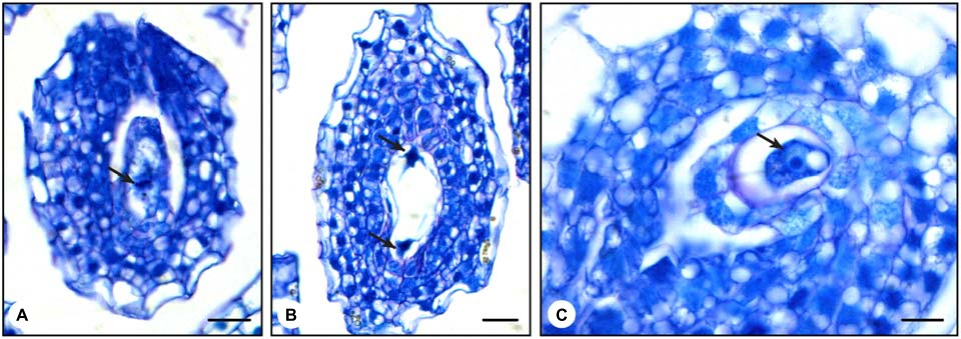
**Histological analysis of the pollination effect on *N. tabacum* late stage 7 ovules. (A)** Ovule from non-pollinated flower bud at late stage 7 (35 mm in size). The picture shows an ovule at the beginning of the first mitotic division, where it is possible to visualize the metaphasic plate (arrow). **(B)** Ovule from non-pollinated flower bud harvested 24 h after being emasculated at late stage 7 (35 mm in size). The picture shows the end of the first mitotic division (arrows). **(C)** Ovule from pollinated flower bud harvested 24 h after pollination at late stage 7 (35 mm in size). This picture shows the egg cell (arrow). This structure is typically observed only in ovules of stage 12 flowers 24 h after anthesis (Tian and Russell, 1997; De Martinis and Mariani, 1999). Bars represent 20 μm in the first two pictures **(A,B)** and 10 μm in the last picture **(C)**.

In *N. tabacum*, the ovules are not fully developed at anthesis (Tian and Russell, 1997; De Martinis and Mariani, 1999). Therefore, how do late stage 7 pollinated pistils produce fruits? To answer this question, we analyzed the effect of pollination on female gametophyte development. Late stage 7 flower buds (35 mm long) were emasculated and either hand pollinated with mature pollen or left unpollinated. This developmental stage was chosen because it is the earliest stage in which fruit formation was observed. After 24 h, the ovaries were collected and prepared for histological analysis. Unpollinated late stage 7 ovaries, 0 h after emasculation, showed young female gametophytes at the beginning of the first mitotic division, in which the formation of the metaphasic plate was observed (**Figure 4A**). In unpollinated late stage 7 ovaries, 24 h after emasculation, we expected to find structures similar to those described in flower buds at stage 8 (De Martinis and Mariani, 1999). As anticipated, it was possible to observe the end of the first mitotic division (**Figure 4B**). In contrast, late stage 7 ovaries 24 h after hand pollination clearly displayed formed egg cells (**Figure 4C**). Under natural conditions, *N. tabacum* egg cells are only found at anthesis or later (Tian and Russell, 1997; De Martinis and Mariani, 1999). Ovule differentiation is not synchronized in *N. tabacum* ovaries, and ovules at the top of the ovary (next to the style) are typically more advanced than ovules at the base of the ovary.

**FIGURE 5:**
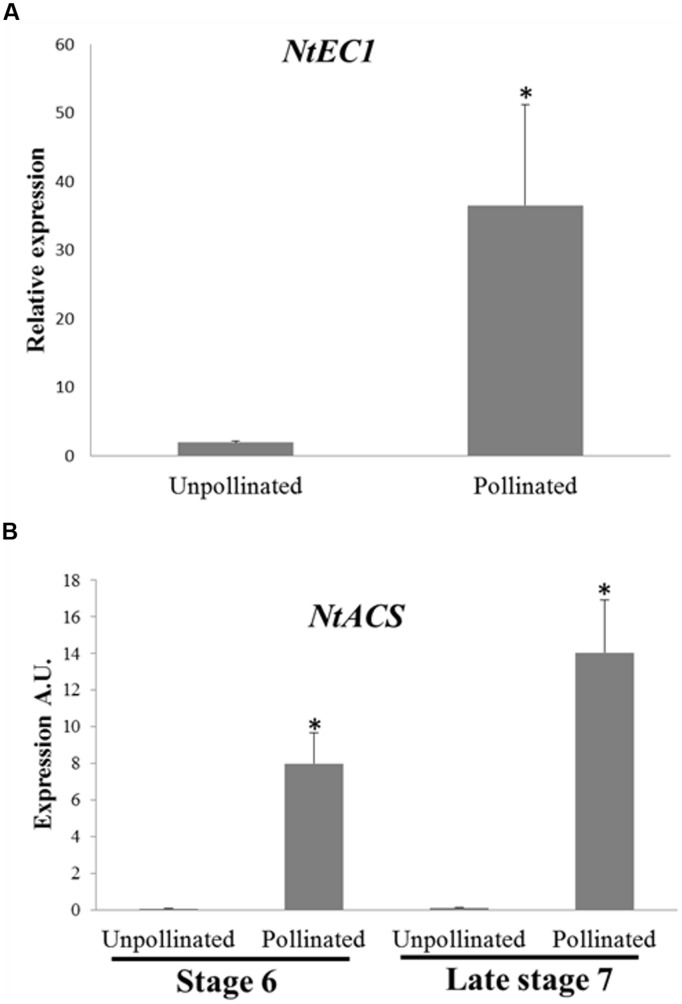
**Expression of *NtEC1* and *NtACS* genes in unpollinated and pollinated pistils of stage 6 and late stage 7 flower buds. (A)** Expression of *NtEC1* gene in unpollinated and pollinated ovaries of late stage 7 flower buds. Expression was normalized using as reference genes GAPDH and RPL2. Relative expression was determined by comparing the *NtEC1* transcript level between unpollinated, considered as 1, and pollinated ovaries. **(B)** Expression of *NtACS* gene in unpollinated and pollinated stigmas/styles of stage 6 and late stage 7 flower buds. The relative expression levels are represented in arbitrary units (A.U.) normalized to the expression level of the GAPDH gene, used as a reference, in each RNA sample. REST statistical analysis indicated a difference in *NtEC1* gene expression among unpollinated and pollinated ovaries **(A)** and differences between unpollinated and pollinated stigma/styles in each stage (6 and late 7; **B)**. (Bars represent the SE and ^∗^ indicates statistically significant difference).

To confirm the effects of pollination on female gametophyte development at the molecular level, we analyzed the expression of the *NtEC1* gene in late stage 7 ovaries 24 h after emasculation (unpollinated) and 24 h after emasculation and pollination (pollinated). *EC1* is a gene specifically expressed in the egg cell (Sprunck et al., 2012; Rademacher and Sprunck, 2013). Therefore, we searched the available databases using the *Arabidopsis EC1.2* sequence (AT2G21740) as a query to find the *N. tabacum EC1* homolog (*NtEC1* – Supplementary Figure S2) and designed specific primers for RT-qPCR. The expression of *NtEC1* was more than 30-fold higher in pollinated compared to unpollinated late stage 7 ovaries (**Figure 5A**). Taken together, these results demonstrate that pollination stimulus is able to accelerate female ovule maturation even in early developmental stages prior to anthesis.

To further clarify the nature of the pollination signal necessary for ovule maturation, we investigated whether pollination would induce ACC synthase expression in young pistils. Flowers at stage 6 and late stage 7 were emasculated and either hand pollinated with mature pollen or left unpollinated. After 24 h, the stigmas/styles were collected and used for RNA extraction. RT-qPCR experiments have shown that unpollinated young pistils do no express *NtACS*, whereas pollination is capable of inducing its expression in stigmas/styles at both developmental stages (**Figure 5B**). There is a difference between *NtACS* expression in pollinated stage 6 and late stage 7 samples; however, this difference is not statistically significant. These results suggest that if ethylene and/or ACC contribute to the pollination signal, they are not sufficient to guarantee successful fertilization and fruit set.

## Discussion

The present study was designed to investigate the developmental timing of important events in preparation for successful fertilization. As a major achievement, we determined the effects of pollination in *N. tabacum* ovule development in flower buds prior to anthesis. At stage 5, the stigma is already receptive, displaying positive peroxidase activity and consistently supporting pollen tube growth through the style. Controlled hand pollinations at different developmental stages demonstrated that fruit formation occurred only at late stage 7 (34–38 mm) onward. Pollinations at stages 5, 6 and early stage 7 did not result in fruits despite stigma receptivity. Based on the developmental and physiological differences concerning fruit formation between 28–33 mm and 34–38 mm stage 7 flower buds, we propose to divide stage 7, previously described by Koltunow et al. (1990), into early stage 7 and late stage 7, respectively.

**FIGURE 6:**
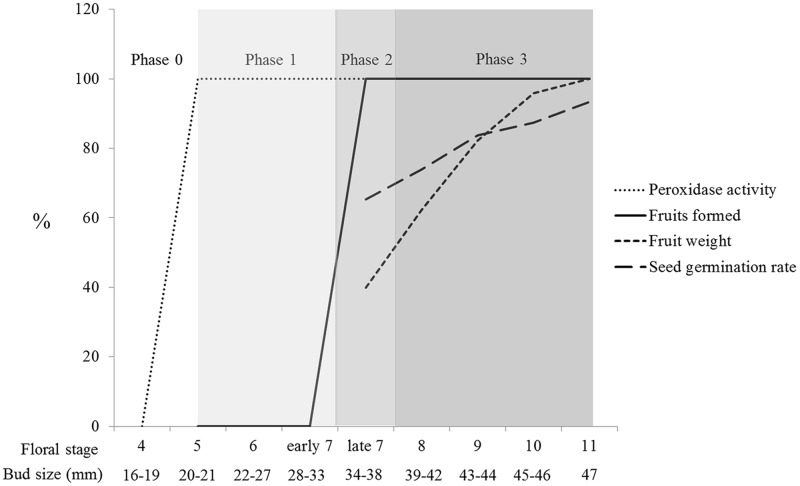
**Phases of tobacco pistil development in preparation for successful pollination and fertilization.** Phase 0 represents the initial developmental stages in which no preparation for pollination exists. Phase 1 was defined as the onset of stigma receptivity (positive peroxidase activity at the stigma surface and sustainable pollen tube growth through the style) until the developmental moment in which pollination results in fruit set. Phase 2 represents the turning point, in which pollinations become productive and are restricted to late stage 7. Phase 3 is comprised of the developmental stages 8–11, in which gradual improvements in fruit weight and seed germination capacity occur.

Effective pollination, which results in fruit set, is mainly determined by stigma receptivity, pollen tube kinetics, and ovule development (Sanzol and Herrero, 2001). Our results on positive peroxidase activity correlated with the stigma’s ability to sustain pollen tube growth or, in other words, stigma receptivity. However, the developmental stages of stigmas/styles influenced the number of growing pollen tubes (**Figure 3**); later developmental stages exhibited higher capacities to sustain a larger number of growing pollen tubes. In some species, the low number of pollen tubes is directly related to fruit abortion (Sutherland, 1987; Björkman, 1995; Niesenbaum, 1999). Our results show that in *N. tabacum*, there is a direct correlation between the developmental stage of the pollinated pistil, the amount of growing pollen tubes, and fruit set (**Figures 3** and **6**; **Table 1**). Therefore, an additional important parameter to be considered for effective pollination is the number of pollen tubes growing through the style.

Which developmental changes in the pistil can affect the number of growing pollen tubes? Pollen tube growth is heterotrophic, and stages 5, 6 and early stage 7 tobacco pistils may not have sufficient carbohydrates to sustain a large number of pollen tubes. An alternative explanation is that styles younger than late stage 7 may not have the necessary intercellular spaces between cells of the transmitting tissue. Tobacco pollen tubes grow in the intercellular spaces of the specialized tissues of the stigma/style, and, at anthesis, these cells are loosely arranged and easily separated (Cresti et al., 1986). The developmentally regulated expression of a pistil-specific pectin acetylesterase gene, which is important for decreasing cell adhesion among these cells (Quiapim et al., 2009) and some other morphological and/or physiological factors, may limit the number of pollen tubes growing in pistils younger than late stage 7. A sharp developmental threshold exists: tobacco flower buds with 33 mm or less in length do not have the necessary characteristics to sustain enough pollen tubes and thus to produce a sufficient number of fertilized ovules. Tobacco flower buds of 34 mm and longer are capable of expressing or have already accumulated all the factors required for growth of a minimum number of pollen tubes and thus for fruit production.

Concerning ovule development, our results clearly show that at late stage 7, ovules are still immature and the female gametophytes are at the beginning of the first mitotic division. Therefore, it is surprising that pollinations at this developmental stage produce fruits and seeds. Our detailed analyses of pollinations performed on late stage 7 pistils demonstrated the effect of accelerating female gametophyte maturation. The pollination effect in triggering ovule maturation was demonstrated both by histological analysis as well as expression of the *NtEC1* gene. In this context, a signal is clearly produced in response to pollen tube growth through the stigma/style and reaches the ovules. What is the nature of this signal? Several types of signals have already been identified as mediators during plant reproduction, such as ethylene (O’Neill et al., 1993; De Martinis and Mariani, 1999; Jones and Woodson, 1999) and its precursor ACC (Jones and Woodson, 1999), gamma-amino butyric acid (Palanivelu et al., 2003), IAA (Chen and Zhao, 2008), jasmonic acid and its derivatives (Avanci et al., 2010; Stitz et al., 2014), calcium (Tian and Russell, 1997; Ge et al., 2009), and peptides (Higashiyama, 2010; Chae and Lord, 2011). Pollination acts as a stimulus and increases the concentration of calcium and ethylene in *N. tabacum* flowers (Tian and Russell, 1997; De Martinis and Mariani, 1999). ACC oxidase-silenced transgenic plants and with impaired ethylene synthesis are unable to complete female gametophyte development (De Martinis and Mariani, 1999). In addition, application of ethylene restores female gametophyte development in these transgenic flowers (De Martinis and Mariani, 1999). Furthermore, the concentrations of enzymes related to ethylene synthesis are altered in response to pollination in Solanaceae species (Llop-Tous et al., 2000; Weterings et al., 2002). These studies were mainly conducted with flowers at developmental stages close to anthesis. However, no detailed study was previously performed to investigate the effect(s) of pollination in young tobacco flower buds, and little is known about the signal(s) produced at these earlier stages.

We investigated the expression of the ACC synthase gene as an attempt to identify the pollination signal that triggers female gametophyte maturation at young pistils. The results show that stage 6 pistils, which are unable to produce fruits, induce *NtACS* expression in a pollination-dependent manner. This result is consistent with the literature (Weterings et al., 2002) and suggests that ACC and/or ethylene is not the primary signal necessary for ovule maturation, at least at early stages of flower development. An alternative explanation is that the ACC and/or ethylene produced at stage 6 does not reach a threshold level and is thus insufficient to trigger the maturation observed at late stage 7. Additionally, ovule maturation is dependent on perception of the pollination signal. Therefore, it is possible that although stage 6 stigmas/styles produce the pollination signal (e.g., ACC and/or ethylene), ovules do not express the signal receptor yet and are incompetent to respond. We remain unable to define the nature of the pollination signal, but it can travel fast and/or act as a long-range signal, reaching embryo sacs located a few centimeters away. Additionally, this pollination signal is so strong that it is capable of overwriting the natural developmental program within the tobacco ovule and accelerates female gametophyte maturation in anticipation of fertilization.

Tobacco pistil development occurs in multiple stage-specific phases along the pistil path (stigma, style, and ovary) in preparation for pollination and fertilization. We propose that phase 0 comprises the initial developmental stages (stages 1–4), from stigma differentiation until the onset of stigma receptivity. Phase 1 represents the first pistil indicator of a preparation for pollination: the onset of peroxidase activity at the stigma surface, which parallels the capacity of sustaining pollen tube growth or stigma receptivity (stages 5 to early stage 7). At this phase, a few pollen tubes reach the ovules but do not penetrate them (Supplementary Figure S1). Phase 2 could be defined as a turning point based on an array of physiological acquisitions that allow a large number of pollen tubes to grow a long distance, reaching, and penetrating the ovules. Thus, late stage 7 corresponds to a critical developmental moment, in which sufficient pollination signal(s) is(are) produced and embryo sacs are capable of perceiving and responding. Hence, female gametophyte maturation is triggered; fruits and seeds are produced. However, the fruits are small, and not all seeds are able to germinate (the germination rate was 65% ± 5.7% for late stage 7 pollination), suggesting that pollination is sufficient but that a parallel mechanism (secondary signals or cellular development) should take place for proper seed formation (stages 11 and 12). Phase 3 is comprised of intermediate developmental stages (stages 8–11) in which gradual improvements take place, resulting in increasingly larger and heavier fruits (which is correlated with the number of seeds), containing seeds with progressively higher germination capacity (**Figure 6**). Phase 4, likely representing the best pollination and fertilization conditions, should occur at anthesis (stage 12), a developmental moment not analyzed in this work.

In recent years, knowledge has increased considerably concerning the responses elicited at the pollen tube in response to its growth along the pistil path and the signals produced by the pistil which are perceived by the pollen (Qin et al., 2009; Palanivelu and Johnson, 2010; Palanivelu and Tsukamoto, 2012; Beale and Johnson, 2013). However, little is known about the pollination signal and the responses triggered at the stigma/style and ovary (the female responses during pollen–pistil interactions). Our results indicate the existence of a powerful program that guarantees the coordinated and synchronized development of male and female gametophytes, ensuring successful reproduction. It would be interesting to perform RNASeq studies to identify genes expressed at different parts of the style, as well as in ovaries, in pollinated, and unpollinated pistils at different developmental stages and at different time points after pollination. These studies would help establish the time necessary for induction/commitment, its physiological basis and, eventually, identification of the pollination signal receptor. Additionally, investigation of the nature of the pollination signal and pistil requirements to perceive it could be useful for the success of self-incompatible and interspecific crosses performed in evolutionary studies and breeding programs.

## Conflict of Interest Statement

The authors declare that the research was conducted in the absence of any commercial or financial relationships that could be construed as a potential conflict of interest.
