# Supplementary material for: Pollination triggers female gametophyte development in immature Nicotiana tabacum flowers
Source: Front Plant Sci. 2015 Jul 22;6:561. doi: 10.3389/fpls.2015.00561 (PMC4510347; doi:10.3389/fpls.2015.00561)
Supplement: Supplementary file 1 [file Presentation_1.PDF]

Supplementary Material

Pollination triggers female gametophyte development in immature *Nicotiana tabacum* flowers

Michael S. Brito[1,2,3,#], Lígia T. Bertolino[1,2], Viviane Cossalter[1], Andréa C. Quiapim[1], Henrique C. DePaoli[1,2,§], Gustavo H. Goldman[4], Simone P. Teixeira[4], and Maria Helena S. Goldman[1*]

[1]Departamento de Biologia, Faculdade de Filosofia, Ciências e Letras de Ribeirão Preto, Universidade de São Paulo, Brazil 14040-901
[2]PPG Genética, Faculdade de Medicina de Ribeirão Preto, Universidade de São Paulo, Brazil; 14049-900
[3]PPG Genética e Melhoramento de Plantas, Faculdade de Ciências Agrárias e Veterinárias, Universidade Estadual Paulista "Júlio de Mesquita Filho," 14884–900 São Paulo, Brazil
[4]Departamento de Ciências Farmacêuticas, Faculdade de Ciências Farmacêuticas de Ribeirão Preto, Universidade de São Paulo, Brazil; 14040-903

*Corresponding author: Prof. Dr. Maria Helena S. Goldman, Departamento de Biologia, Faculdade de Filosofia, Ciências e Letras de Ribeirão Preto, Universidade de São Paulo, Avenida Bandeirantes, 3900. Ribeirão Preto, SP - CEP 14040-901, BRAZIL. Phone: 55-16-3315-3702; FAX: 55-16-3315-4886 ; e-mail: mgoldman@ffclrp.usp.br

1. Supplementary Figures

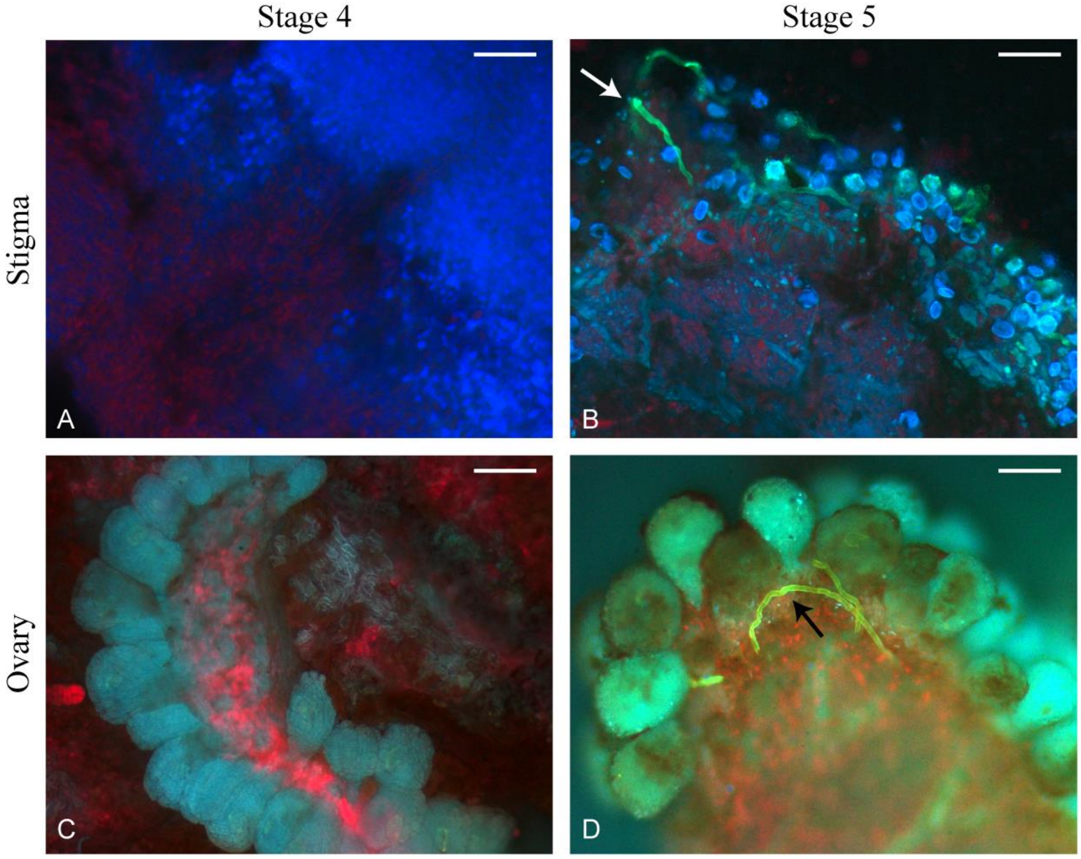

Supplementary Figure 1 – Pollen tube germination and growth after pollinations performed at stages 4 and 5 of *N. tabacum* flower development. A) Stage 4 stigma: pollen grains hardly stick on the stigma surface and do not germinate. **B)** Stage 5 stigma: the first developmental stage in which pollen grains germinate on the stigma surface. **C)** Stage 4 ovary: consistently with what was observed at the stigma surface, it is not possible to observe any pollen tube growing in the ovary at this stage. **D)** Stage 5 ovary: pollen tubes clearly grow close to the ovules. Arrows indicate germinated pollen grains and growing pollen tubes. Scale bars represent 100 µm.

Material & Methods - Controlled hand pollinations were done at stages 4 and 5 *N. tabacum* pistils with mature pollen from stage 12 flowers. After 24 hours, samples were prepared for visualization, separating the stigmas/styles and ovaries. Both pistil parts were placed on histological glass slides and stained with a solution of 0.05% of aniline blue (Merck), diluted in potassium phosphate buffer pH 8.5 (Spence, 2001). The plant material was then carefully squashed between glass slide and coverslip in the aniline blue solution, revealing the pollen tube callose plugs. The visualization and documentation were performed in a Zeiss Axiolab epifluorescence microscope (HBO 103W/2 lamp), using an excitation wavelength of 450/90nm and an emission wavelength of 520nm. Images were taken using a Zeiss AxioCam Color 412-312 and AxioVision LE4.8 software.

Spence, J. (2001). Plant histology. In: Plant Cell Biology – Second Edition. Hawes, C. and Satiat-Jeunemaitre, B. (eds). Oxford University Press.

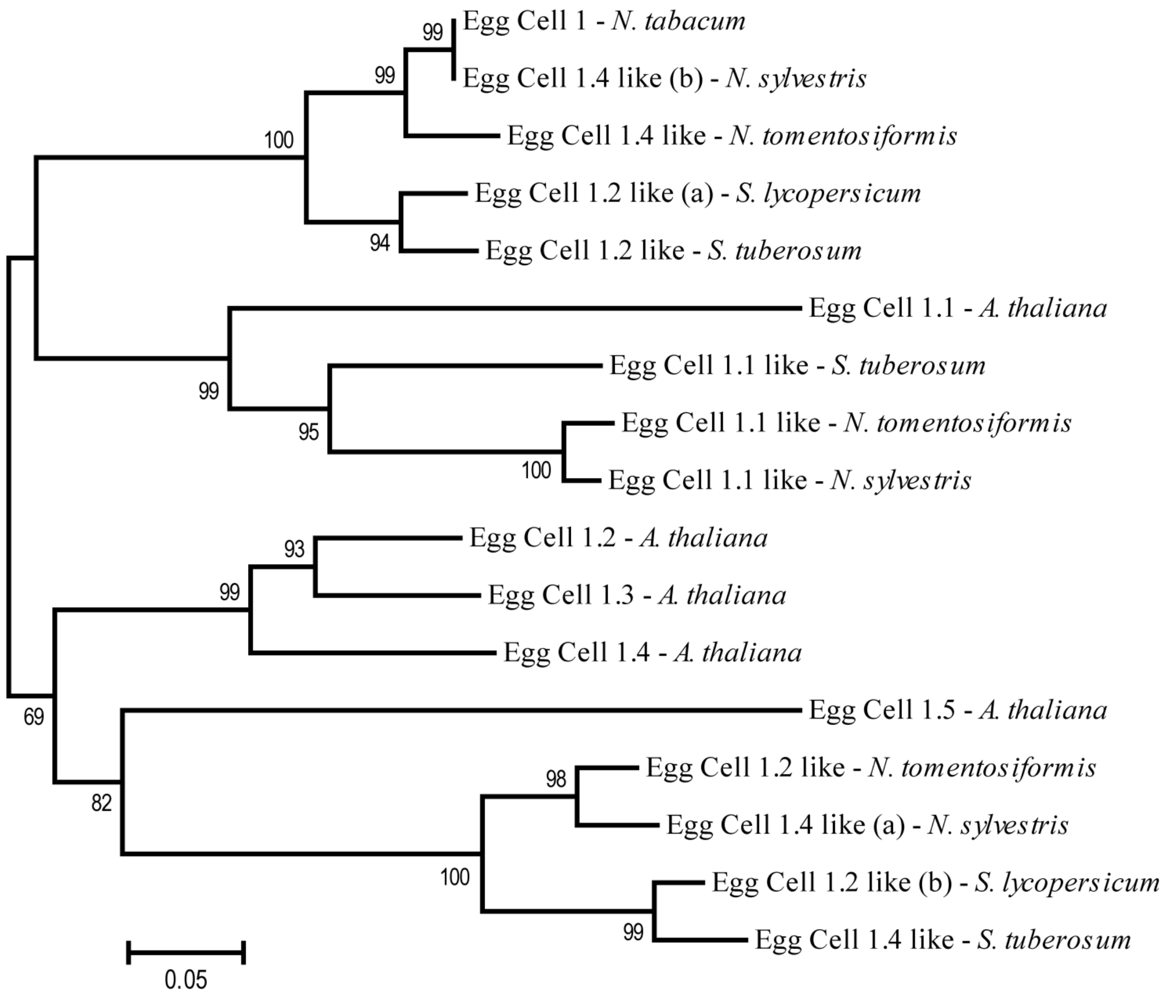

Supplementary Figure 2 - Neighbor-Joining tree obtained from analysis of Egg Cell 1 amino acid sequences from *Arabidopsis thaliana*, *Solanum lycopersicum*, *Solanum tuberosum*, *Nicotiana sylvestris*, *Nicotiana tomentosiformis* and *Nicotiana tabacum*. Bootstrap values higher than 50% are shown for 1000 replicates. The bar indicates number of substitutions per site.

Material & Methods - Amino acid sequences were aligned with Muscle. The topology was obtained by Neighbor-Joining method with genetic distances calculated by p-distance method using 1000 bootstrap replicates. The evolutionary analyses were conducted in MEGA5. Sequences from *Arabidopsis thaliana* used in this analysis are available in TAIR (https://www.arabidopsis.org) under the following accession numbers: Egg Cell 1.1 - AT1G76750; Egg Cell 1.2 - AT2G21740; Egg Cell 1.3 - AT2G21750; Egg Cell 1.4 - AT4G39340; Egg Cell 1.5 - AT5G64720. Sequences from *Solanum lycopersicum*, *Solanum tuberosum*, *Nicotiana sylvestris*, *Nicotiana tomentosiformis* and *Nicotiana tabacum* are available in NCBI (http://www.ncbi.nlm.nih.gov) under the following accession numbers: Egg Cell 1.2 like (a) - *S. lycopersicum* - XM_010321796.1; Egg Cell 1.2 like (b) - *S. lycopersicum* - XM_010314587.1; Egg Cell 1.1 like - *S. tuberosum* - XM_006350983.1; Egg Cell 1.2 like - *S. tuberosum* - XM_006367743.1; Egg Cell 1.4 like - *S. tuberosum* - XM_006350982.1; Egg Cell 1.1 like - *N. sylvestris* - XM_009768422.1; Egg Cell 1.4 like (a) - *N. sylvestris* - XM_009781801.1; Egg Cell 1.4 like (b) - *N. sylvestris* - XM_009790265.1; Egg Cell 1.1 like - *N. tomentosiformis* - XM_009591597.1; Egg Cell 1.2 like - *N. tomentosiformis* - XM_009591598.1; Egg Cell 1.4 like - *N. tomentosiformis* - XM_009611321.1; Egg Cell 1 – *N. tabacum* - KP987452.

2. **Supplementary Tables**

Supplementary Table 1. REST statistical analyze comparing *NtEC1*expression in pollinated and unpollinated ovaries at late stage 7 flower buds. Relative expression report.

Parameter	Value
Iterations	6000

Gene	Type	Reaction Efficiency	Expression	Std. Error	95% C.I.	P(H1)	Result
GAPDH/ RPL2	REF	1.0	1.000	-	-	-	-
NtEC1	TRG	1.0	7.968	2,565 - 16,661	1,591 - 32,657	0,000*	UP[1]

TRG, Target. REF, Reference. P(H1), Probability of alternate hypothesis that difference between sample and control groups is due only to chance.
* *NtEC1* expression is different in pollinated compared to unpollinated group, P(H1)=0.000.
[1] *NtEC1* is UP-regulated in sample group (in comparison to control group) by a mean factor of 7.968 (S.E. range is 2.565 – 16.661).

Supplementary Table 2. REST statistical analyze comparing *NtACS* expression in pollinated and unpollinated stigma/styles from stage 6 (A), late stage 7 (B) and pollinated stigma/styles from late stage 7 and stage 6 (C).

A) Comparison among pollinated and unpollinated stigma/styles from flower buds of stage 6. Relative expression report.

Parameter	Value
Iterations	6000

Gene	Type	Reaction Efficiency	Expression	Std. Error	95% C.I.	P(H1)	Result
GAPDH	REF	1	1	-	-	-	-
NtACS	TRG	1	202.683	63.597 - 959.387	56.544 - 2028.474	0.002*	UP[1]

TRG, Target; REF, Reference; P(H1), Probability of alternate hypothesis that difference between sample and control groups is due only to chance.
* *NtACS* sample group is different to control group, P(H1)=0.002.
[1] *NtACS* is UP-regulated in sample group (in comparison to control group) by a mean factor of 202.683 (S.E. range is 63.597 – 959.387).

B) Comparison among pollinated and unpollinated stigma/styles from flower buds of late stage 7. Relative Expression report.

Parameter	Value
Iterations	6000

Gene	Type	Reaction Efficiency	Expression	Std. Error	95% C.I.	P(H1)	Result
GAPDH	REF	1	1	-	-	-	-
NtACS	TRG	1	104.071	52.988 – 206.530	32.946 – 210.167	0.003*	UP[1]

TRG, Target; REF, Reference; P(H1), Probability of alternate hypothesis that difference between sample and control groups is due only to chance.
* *NtACS* sample group is different to control group, P(H1)=0.003.
[1] *NtACS* is UP-regulated in sample group (in comparison to control group) by a mean factor of 104.071 (S.E. range is 52.988 – 206.530).

C) Comparison among pollinated stigma/styles from flower buds of late stage 7 and stage 6. Relative Expression report.

Parameter	Value
Iterations	6000

Gene	Type	Reaction Efficiency	Expression	Std. Error	95% C.I.	P(H1)	Result
GAPDH	REF	1	1	-	-	-	-
NtACS	TRG	1	1.682	0.977 – 3.375	0.462 – 3.435	0.092*	-

TRG, Target; REF, Reference; P(H1), Probability of alternate hypothesis that difference between sample and control groups is due only to chance.
* *NtACS* sample group is not different to control group, P(H1)=0.092.
